# Multi-virulence of *Campylobacter jejuni* carried by chicken meat in Brazil

**DOI:** 10.3389/fmicb.2023.1220579

**Published:** 2023-08-04

**Authors:** Phelipe Augusto Borba Martins Peres, Roberta Torres de Melo, Paulo Marcel Armendaris, Fabiano Barreto, Tiago Follmann Perin, Ana Laura Grazziotin, Guilherme Paz Monteiro, Ana Beatriz Garcez Buiatte, Eliane Pereira Mendonça, Eduarda Cristina Alves Lourenzatto, Artur Slompo Muniz Bicalho, Marcelo de Vito Filho, Daise Aparecida Rossi

**Affiliations:** ^1^Laboratório de Epidemiologia Molecular - LEPIMOL/UFU, Uberlândia, Minas Gerais, Brazil; ^2^Laboratório Federal de Defesa Agropecuária/RS - LFDA/RS, Porto Alegre, Rio Grande do Sul, Brazil

**Keywords:** poultry-farm, public health, environmental adaption, multi-virulence, MALDI-TOF

## Abstract

*Campylobacter jejuni* is the most frequent cause of bacterial gastroenteritis; therefore, the characteristics of its epidemiology must be continuously investigated to support possible mitigating measures. This is particularly important when evaluating representative strains from the world's leading chicken meat exporter, Brazil. We evaluated a panel of 14 virulence genes in 359 strains of *C. jejuni* isolated from chilled broiler carcasses in Brazil. The genes were classified into five virulence categories (B: biofilm/motility; SS: secretion/cytotoxicity system; CI: invasion/colonization; GB: Guillain-Barré; and AE: adaptation to stress). The percentage of strains with stress adaptation genes (86.07%) indicates the ability to survive in unfavorable environments; in addition, the strains showed a risk of causing infections in humans due to the frequency of the hcp gene (97.77%). Genes related to Guillain-Barre syndrome (GBS) in 77.44% of strains are an additional concern, which must be monitored. The gene panel showed the presence of 124 virulence profiles. Individual analyses by carcass, slaughter establishment, and municipalities in which they were located showed high index variabilities (I.Var.) of 0.82, 0.87, and 0.78, respectively. Georeferencing indicated the state of Paraná as a hotspot for virulent strains. Higher levels of isolation and multi-virulence were identified in the summer, which is hot and humid in Brazil. Together, our results showed that the studied strains are a potential danger to public health and that there is an urgent need for their surveillance and the adoption of control measures, especially in the state of Paraná.

## 1. Introduction

*Campylobacter jejuni* is one of the most frequent causes of bacterial gastroenteritis worldwide, being the most prevalent agent in countries with structured surveillance systems (CDC, 2019; EFSA, 2020). In low- and middle-income countries (LMICs), there is underreporting of human infections and the identification of contaminated food. These problems, added to the multiplicity of reservoirs, transmission routes, and geographical and seasonal distribution, make it difficult to fully understand its epidemiology (Kaakoush et al., 2015).

This microorganism is considered fastidious, has a high potential to form biofilms, easily acquires the viable non-cultivable form under conditions of extreme stress, and has a variety of virulence factors. These genetic determinants guarantee not only efficiency in the infectious processes in the human host but also survival in hostile environments. They are fundamental to the characterization of different strains (Ungureanu et al., 2019).

Some genes have been recognized for their importance in the genus *Campylobacter*, including *flaA* and *luxS* involved in the formation of biofilms, *cdt*ABC and hcp related to secretion systems, *cadF, ciaB*, and *pldA* related to invasion and colonization, *dnaJ, htrA*, and *cbrA* involved in adaptation to environmental stress, and *neuA* and *cstII* related to the induction of Guillain-Barré Syndrome (Amon et al., 2012; Bolton, 2015; Melo et al., 2017; Serichantalergs et al., 2020).

Given this, conducting epidemiological analysis becomes a difficult and, at the same time, fundamental task in adopting intervention measures for its control and prevention (Llarena et al., 2017). These analyses are particularly relevant in the poultry industry since the consumption of chicken meat contaminated with *Campylobacter* is one of the main sources of infection for humans, and Brazil is the world's largest exporter of this food (Omurtag et al., 2013; ABPA, 2023). This study aimed to determine the virulent genetic potential and genotypic spread of *C. jejuni* isolated from broiler carcasses destined for internal and external trade. In this study, it was possible to develop an index for the classification of virulent and multi-virulent strains, aiming to assist further epidemiological studies of the species.

## 2. Methodology

### 2.1. Collection, isolation, and identification of *C. jejuni*

Overall, 359 strains of *C. jejuni* isolated from 114 refrigerated chicken carcasses, slaughtered in establishments registered with the Federal Inspection Service by the Ministry of Agriculture, Livestock, and Supply (MAPA), were analyzed. The carcasses were analyzed from October 2017 to July 2018 to meet the Exploratory Program for the Research and Estimation of the Prevalence of *Campylobacter* spp., the same ones used for the official verification of the *Salmonella* spp. Control and Monitoring Program, determined by IN n° 20 of 21 October 2016 (MAPA, 2016). The collections of carcasses were carried out in establishments located in the states of Paraná (A), Santa Catarina (B), and Rio Grande do Sul (C), distributed across 43 municipalities (Rodrigues et al., 2021).

The strains were isolated and identified as *C. jejuni* in official MAPA laboratories using the methodology proposed by ISO 10272-1: 2017 (ISO, 2017) and mass spectrometry (MALDI TOF^®^) for the identification of the genus and species, respectively. Additional information, such as establishment and place and date of isolation, was received together with the cryopreserved strains and deposited in the culture bank of the Molecular Epidemiology Laboratory of the Faculty of Veterinary Medicine of the Federal University of Uberlândia (LEPIMOL-FAMEV-UFU).

The samples were prepared according to the direct transfer protocol (Direct Transfer Method) for the plate from the isolated colony in three spots. After application to the plate, the sample was covered with 1 μl of alpha-cyano-4-hydroxycinnamic acid (HCCA) solution. Reference strains belonging to LANAGRO-RS were used as controls. The Brucker platform, model Autoflex Speed, was used to identify the isolates. The spectrum bank was the MALDI Biotyper RTC/OC 3.1. The species decision criteria are as follows: equal to or >1.7 for genus and equal to or >2.0 for genus and species. The data acquisition control program was Brucker FlexControl 3.4. Calibration was performed using the Protein Standard I calibrator, with a calibration error tolerance of <200 ppm.

### 2.2. Virulence panel

After reactivation on Campylobacter Blood-Free Selective Agar (mCCDA) and incubation in a microaerobic atmosphere (5–15% O_2_ and 10% CO_2_) using a Microaerobac (Probac do Brasil, São Paulo, Brazil) at 37°C for 44 ± 4 h, followed by strain confirmation by oxidase, catalase, motility, and typical morphology tests (ISO, 2017), genomic DNA was extracted using the Wizard Genomic DNA Purification Kit (Promega, cat no. 0000110703, São Paulo, Brazil), following the protocol provided by the manufacturer. Purified DNA (10 ng) was used as a template for all PCR reactions. The PCR conditions and primers used in this study are described in Table 1.

**Table 1:** PCR conditions, nucleotide sequences, and sizes of amplicons to identify virulence genes in *C. jejuni*.

**Virulence category**	**Gene**	**Amplicon (bp)**	**Primer/Sequence 5^′^ → 3^′^**	**Volume/DNA/ Primer/Annealing**	**References**
Biofilm formation	*flaA*	1728	F-ATGGGATTTCGTATTAACAC R-CTGTAGTAATCTTAAAACATTTTG	25 μl/20 ng/10 pmol/45°C-1 min	Hänel et al., 2004
	*luxS*	1080	F-AGGCAAAGCTCCTGGTAAGGCCAA R-GGATCCGTATAGGTAAGTTCATTTTTGCTCC	25 μl/50 ng/10 pmol/55°C-1 min	Elvers and Park, 2012
Secretion systems	*cdtA*	420	F-CTATTACTCCTATTACCCCACC R-AATTTGAACCGCTGTATTGCTC	25 μl/80 ng/10 pmol/57°C-1 min	Martínez et al., 2006
	*cdtB*	531	F-AGGAACTTTACCAAGAACAGCC R-GGTGGAGTATAGGTTTGTTGTC		
	*cdtC*	339	F-ACTCCTACTGGAGATTTGAAAG R-CACAGCTGAAGTTGTTGTTGGC		
	*hcp*	510	F-TGGCTGAACCAGCGTTTATAAAAATTG R-TTAAGCTTTGCCCTCTCTCCA	25 μl/20 ng/10 pmol/57°C-30 s	Singh et al., 2019
Invasion/ Colonization	*cadF*	400	F-TTGAAGGTAATTTAGATATG R-CTAATACCTAAAGTTGAAAC	25 μl/20 ng/40 pmol/47°C-1 min	Zheng et al., 2006
	*ciaB*	527	F-TGCGAGATTTTTCGAGAATG R-TGCCCGCCTTAGAACTTACA	25 μl/20 ng/10 pmol/45°C-1 min	
	*pldA*	385	F-AAGAGTGAGGCGAAATTCCA R-GCAAGATGGCAGGATTATCA		
Adaptation to stress	*dnaJ*	720	F-AAGGCTTTGGCTCATC R-CTTTTTGTTCATCGTT	25 μl/20 ng/10 pmol/46°C-1 min	Datta et al., 2003
	*htrA*	1393	F-TAATACGACTCACTATAGGGTAAGTTTAG CAAGTGCTTTATTTGC R-AAAACCATTGCGATATACCCAAACT		
	*cbrA*	1165	F-TAATACGACTCACTATAGGGTCAACTCTA TCCTTGCCATTATCTT R-GTAGATATTGCTTTTGGTTTTGCTG	25 μl/20 ng/50°C-1 min	Biswas et al., 2011
GBS induction	*cstII*	400	F-GTTATTATTGCTGGAAATGGACCAAGT R-ACATATAGACCCCTGAGGTAATTCTTT	25 μl/20 ng/10 pmol/52°C-1 min	Amon et al., 2012
	*neuA*	500	F-GCTCGTGGTGGCTCAAAGGG R-ATTGCACCATTGCTCATATA		

Overall, 14 genes were studied and divided into five virulence categories: i) biofilm formation – *flaA* (motility) and *luxS* (quorum-sensing mechanism); ii) secretion systems – *cdt*ABC (distensive cytotoxin secretion) and *hcp* (type VI secretion system); iii) invasion and colonization – *cadF* (intracellular colonization), *ciaB* (cell invasion), and *pldA* (invasion/colonization); iv) adaptation to stress – *dnaJ* (thermotolerance), *htrA* (aid in growth under stress), and *cbrA* (resistance to osmotic shock); and v) induction of Guillain-Barré Syndrome – *cstII* (GBS) and *neuA* (GBS).

PCR reactions were performed using the GoTaq^®^ Green Master Mix kit (Promega, cat no. 0000112705, São Paulo, Brazil), according to the manufacturer's instructions. As positive controls, strains of *C. jejuni* ATCC 33291, IAL 2383, and NCTC 11351 were used. The amplified products were subjected to electrophoresis in a 1.5% agarose gel, using the TBE 0.5× running buffer (Invitrogen) and, as a molecular weight standard, the 100 bp marker (Invitrogen).

A criterion adapted from the analysis of the antimicrobial multi-resistance index (Biswas et al., 2011) and the five categories described in Table 1 were used to classify the virulence level of the strains. Thus, the strains were classified as virulent (V) when they had at least one gene from each category and multi-virulent (MV) when they had two or more genes from each category.

The variability of the strains within a given group (sample, establishment, municipality, or state) was verified using the analysis of the number of distinct virulence profiles identified. The variability, virulence, and multi-virulence indices were determined by calculating the relative frequency for each group of strains evaluated. The classification for the variability index was adapted (Krumperman, 1983), being low for values <0.5, medium for values between 0.5 and 0.7, and high for values >0.7.

### 2.3. Statistical analysis

The analysis of the results of the virulence panel was performed based on the number of strains isolated in each analyzed sample, the establishment of origin, the place (city and state), and the seasonal aspects for the percentage description. For comparative analyses, the normality of the data was verified, followed by the application of Fisher's exact test or a Student's *t*-test to compare two variables and ANOVA or Kruskal–Wallis in the comparison of three or more variables. The program used was GraphPad Prism 8.0.1 with a 95% confidence interval.

## 3. Results

### 3.1. Genes, virulence profiles, and isolate analysis by sample

A total of 14 genes associated with virulence were studied in all 359 strains of *C. jejuni* isolated from chicken carcasses. The evaluation by virulence category found that the concomitant frequency of genes linked to adaptation to stress was significantly higher (86.07%; 309/359) compared to the remaining categories, and those related to secretion were the least frequent (131/359; 36.49%) (Table 2). The majority of the isolates (292/359; 81.34%) had at least eight of the 14 studied genes (*p* < 0.05, Fisher's test), which portrayed the high percentage of virulent strains (229/359; 72.14%), of which 47.60% (109/229) were classified as multi-virulent. We found a total of 124 virulence profiles, of which five represented 40.1% (144/359) of the strains, with 76 strains belonging to a single multi-virulent profile (*cdtA, cdtB, cdtC, luxS, pldA, flaA, dnaJ, cadF, neuA, ciaB, cstII, cbrA, htrA*, and *hcp*). This fact determined the low variability index (I.Var. = 0.35) considering the 359 strains.

**Table 2:** Frequency of virulence genes in *C. jejuni* isolated from chicken carcasses in Brazil between October 2017 and July 2018.

**Virulence categories**	**Gene**	**Gene frequency (%)**	**Category frequency (%)**
Adaptation to stress	*cbrA*	346 (96.38)	309 (86.07)^a^
	*htrA*	338 (94.15)	
	*dnaJ*	326 (90.81)	
Guillain-Barré Syndrome	*neuA*	319 (88.86)	268 (74.65)^b^
	*cstII*	278 (77.44)	
Invasion/colonization	*cadF*	300 (83.57)	229 (63.79)^c^
	*ciaB*	295 (82.17)	
	*pldA*	262 (72.98)	
Biofilm/motility	*luxS*	236 (65.74)	182 (50.70)^d^
	*flaA*	201 (55.99)	
Secretion system/cytotoxicity	*hcp*	351 (97.77)^*^	131 (36.49)^e^
	*cdtA*	170 (47.35)	
	*cdtB*	151 (42.06)	
	*cdtC*	162 (45.13)	

Different letters in the column indicate a significant difference.

* p < 0.0001 in the analysis within the same category.

Profiles including genes linked to the biofilm formation/motility category were the least identified (60.5%; 75/124), and those associated with secretion systems (95.2%; 118/124) and adaptation to stress (96.0%; 119/124) were the most prevalent. A total of 16 clusters (A1 to A16) of virulence categories were identified, with A16 comprising 57 profiles (46%), of which 14 were classified as multi-virulent (Table 3).

**Table 3:** Virulence profiles classified according to the categories of studied genes in *C. jejuni* isolated from chicken carcasses in Brazil between October 2017 and July 2018.

**Category**	**Number of profiles**
Biofilm (B)	75^c^ (60.5%)
Secretion system (SS)	118^b^ (95.2%)
Invasion/colonization (IC)	106^a^ (85.5%)
Guillain-Barré Syndrome (GB)	102^a^ (82.3%)
Adaption to stress (AE)	119^b^ (96.0%)
**Grouping**	**Number of profiles**
A1. SS	2 (1.61%)
A2. GB	1 (0.80%)
A3. B, AE	1 (0.80%)
A4. GB, AE	1 (0.80%)
A5. IC, AE	1 (0.80%)
A6. SS, AE	3 (2.41%)
A7. B, GB, AE	1 (0.80%)
A8. B, SS, IC	1 (0.80%)
A9. B, SS, GB	1 (0.80%)
A10. B, IC, AE	1 (0.80%)
A11. SS, IC, AE	2 (1.61%)
A12. SS, GB, AE	6 (4.83%)
A13. B, SS, GB, AE	2 (1.61%)
A14. B, SS, IC, AE	11 (8.87%)
A15. SS, IC, GB, AE	33 (26.61%)
A16. B, SS, IC, GB, AE (Multi-virulent)	**57** ^*^ **(46.0%)**
Total	**124 (100%)**

A: grouping. Different letters in the column indicate a significant difference.

* Significantly different from others. P < 0.05.

GB, Guillain-Barré syndrome; AE, adaptation to stress; IC, cell invasion/colonization; SS, secretion system/cytotoxicity; B, biofilm/motility.

**Figure 1:**
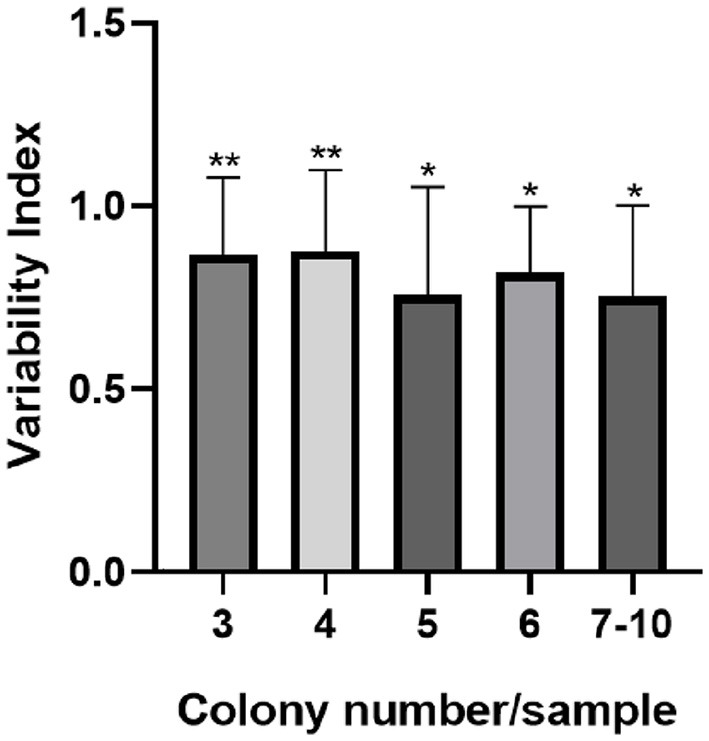
Variability index of *C. jejuni* per sample according to the number of isolated colonies. The error bar indicates the standard deviation, *p < 0.05; **p < 0.01 by the Kruskal–Wallis test.

The 359 strains used in our study came from the analysis of 114 carcasses, of which one to ten distinct colonies of *C. jejuni* were isolated per sample (Md = 3). Samples in which we obtained isolates from three or more colonies (59/114; 51.75%) were grouped according to the number of isolates to determine the variability index (I.Var.), whose value identified did not differ between groups (*p* = 0.4841). The elevated I.Var. average found (0.82) justified the individual analysis of each strain throughout the study. The I.Var. identified within each group differed significantly (*p* < 0.05), and this value was >0.75 for all samples (Figure 1).

Our study included the analysis of 46 slaughterhouses under federal supervision in 43 municipalities. We obtained a variation of one to 22 isolates in each location, and those establishments that presented three or more strains (36/46; 78.3%) were used to determine the general and individual I.Var. scores. In the interpretation of virulence and multi-virulence indices, all establishments were considered.

The average I.Var. score of all establishments was 0.87, with the majority of them (28/36; 77.78%) showing values above 0.7 (Table 4).

**Table 4:** Variability index of virulence profiles of *C. jejuni* isolated from chicken carcasses by slaughterhouse.

**I.Var. of virulence profiles**	**Classification of I.V**.	**Establishments (*n* = 36)**	**Total**
1	High	10 (27.78%)^ab^	28 (77.78%)^a^
0.99–0.7	High	18 (50.00%)^a^	
0.69–0.5	Medium	7 (19.44%)^bc^	8 (22.22%)^b^
< 0.5	Low	1 (2.78%)^c^	

Different letters in the same column indicate a significant difference by Fisher's exact test (p < 0.05).

The distribution of establishments according to the classification of virulence and multi-virulence is described in Table 5. In general, and according to our expectations, the number of virulent strains per establishment and between establishments was significantly higher than that of multi-virulent strains (Student's *t*-test, *p* < 0.001). The significant majority of establishments (21/46; 45.7%) had 70–100% of the strains classified as virulent, and two had the same percentage range of multi-virulent strains, both located in state A. Percentages < 40% of virulent and multi-virulent strains were identified in 11/46 (23.9%) and 33/46 (71.8%) of the establishments, respectively.

**Table 5:** Frequency distribution of virulent and multi-virulent strains by establishment.

**Percentage range of strains/establishments with the characteristics (%)**	**Establishments with virulent strains [n (%)]**	**Establishments with multi-virulent strains [*n* (%)]**
100–70	21 (45.7)^a, A^	2 (4.3)^b, A^
70–40	14 (30.4)^a, AB^	11 (23.9)^a, B^
40–0	11 (23.9)^a, B^	33 (71.8)^b, C^
Total	46	46

Different lowercase letters on the same line and different uppercase letters in the same column indicate a significant difference (p < 0.05).

### 3.2. Analysis of isolates by state, municipality, and seasonality

The characteristics of *C. jejuni* were analyzed according to their states of origin: A, B, and C. State A had the lowest number of isolates (81/359; 22.6%) and profiles (42/124; 33.9%) compared to the other two states (*p* < 0.05), whose numbers were similar (Table 6).

**Table 6:** Variability indices, strain frequencies, and virulence profiles identified in *C. jejuni* isolated from chicken carcasses in Brazil.

		**A**	**B**	**C**
I.Var. (classification)		0.53 (medium)	0.42 (low)	0.51 (medium)
Profiles with 1 strain [*n* (%)]		30 (71.4)aA	35 (64.8)^aA^	53 (69.7)^aA^
Profiles with 2–3 strains [*n* (%)]		10 (23.8)^aB^	15 (27.8)^aB^	16 (21.1)^aB^
Profiles with more than 3 strains [*n* (%)]		2 (4.8)^ab^	4 (7.4)^Ab^	7 (9.2)^aB^
Total	Strains (*n*)	81^a^	128^b^	150^b^
	Profiles (n)	42^a^	54^b^	76^b^

Different lowercase letters in the same row and different uppercase letters in the same column indicate a significant difference; (p < 0.05) - One way ANOVA and Fisher's test. I.Var., index of variability; n (%), frequency and percentage of characteristic.

**Figure 2:**
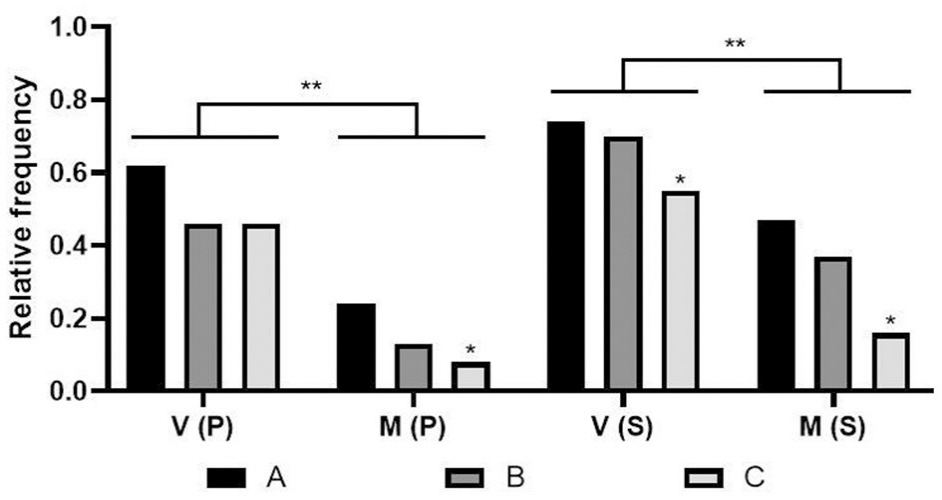
Relative frequency of virulent and multi-virulent strains by state. V: Virulence; M: Multi-virulence; P: profiles; S: strains; States: A, B, and C; *p < 0.05; **p < 0.005 - One way ANOVA and Fisher's test.

Despite presenting more isolates, state C had the lowest relative frequency (Fisher's test, *p* < 0.05) of strains classified as virulent and multi-virulent compared to states A and B, as well as multi-virulent profiles (Figure 2).

**Figure 3:**
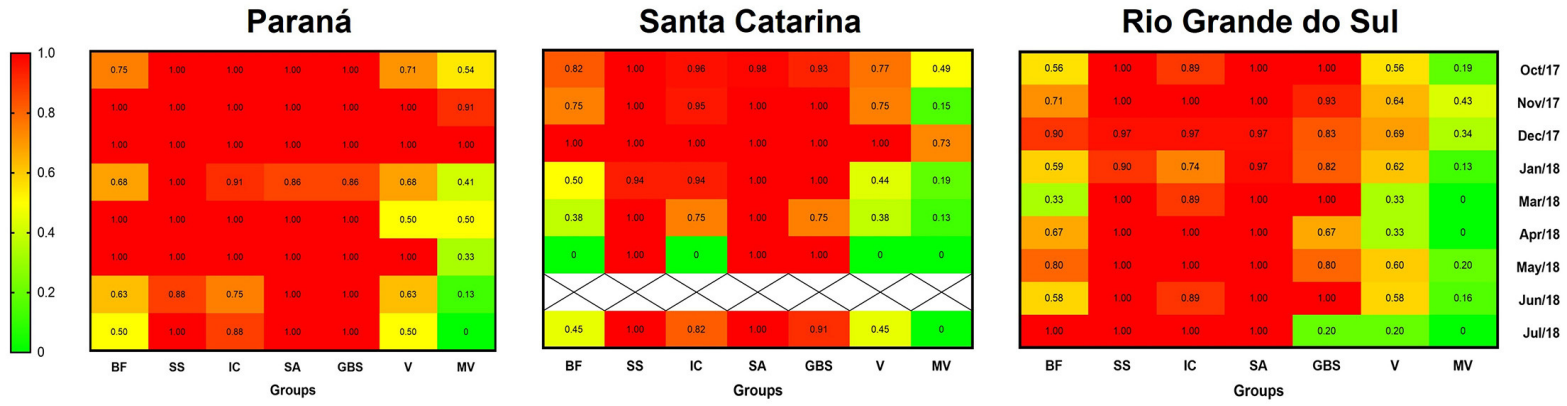
Heat graph based on the extreme colors from green to red, which indicates the relative frequency of each virulence category studied according to the state and months of isolation of *C. jejuni*. BF, Biofilm formation group (frequency of one or more genes); SS, Secretion system (frequency of one or more genes); IC, Invasion/Colonization (frequency of one or more genes); SA, Stress adaptation (frequency of one or more genes); GBS, Guillain-Barré syndrome (frequency of one or more genes); V, index of virulent strains; MV, index of multi-virulent strains; X, absence of isolates (GraphPad Prism 8.0.1).

The construction of the heat graph led to the identification of more evident hot zones in state A. Genes linked to biofilm formation were the least frequent but predominant in all states in December and most common in state A. The highest concentration of virulent and multi-virulent strains was also more expressive in state A, especially in November and December. Thus, state A presents itself as the main hotspot in terms of potential for the maintenance of virulent and multi-virulent strains of *C. jejuni* compared to the other states. The other genetic categories (SS, IC, and SA) showed a similar frequency in the three states (Figure 3).

Even with a significant number of profiles that contemplate only one strain in the three states, no state had a high I.Var. due to the large number of strains being grouped into a few profiles (Table 6). In state C, the variability below 0.7 is due to the existence of two profiles comprising 16 and 18 strains. In state B, the lower value is linked to a profile that includes 37 multi-virulent strains, and in state A, to a multi-virulent profile with 25 strains.

**Figure 4:**
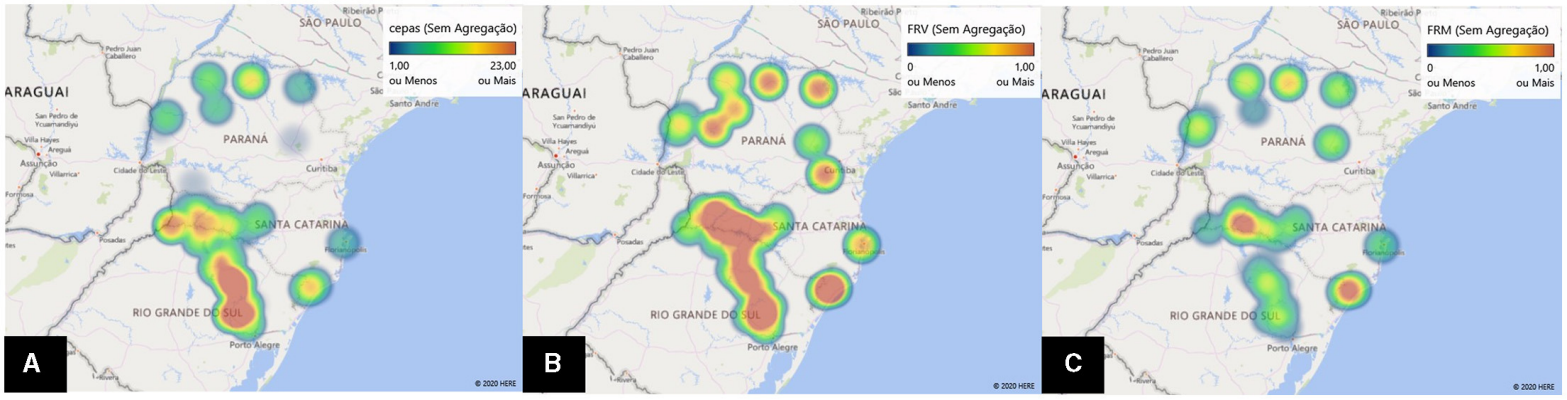
Heat maps. Distribution of the number of *C. jejuni* isolates **(A)**, relative frequency of virulent **(B)**, and multi-virulent **(C)** strains from chicken carcasses in southern Brazil (Microsoft Office – Excel 365, 2016).

The georeferencing of the strains made it possible to determine the specific regions within each state where there is greater densification in terms of the number of isolates (Figure 4A). High relative frequencies of virulent (Figure 4B) and multi-virulent (Figure 4C) strains were evident in the geocoded hot tags.

In state A, the distribution of the number of isolated strains showed a more dispersed characteristic, but the greatest densification came from the north, northwest, and west regions. Paradoxically, this high dispersion masks its high rates of virulence and multi-virulence, especially in the central and metropolitan regions (Figure 4).

States B and C had a high and significant number of isolates, with a more convergent disposition. In Santa Catarina, the predominance was evident in the western, plateau, and southern regions. In Rio Grande do Sul, the strains were found exclusively in the northwest, northeast, and central-east regions of the state. Virulence characteristics are evident in both states, but the hot zones referring to the multi-virulence index are restricted only to the west and south of SC (Figure 4).

There was a variation of one to 23 isolates per city, with a median of eight isolates. To determine the I.Var. and the distribution of virulence profiles according to the classification of V and MV, the selected municipalities were those that presented three or more isolates, which corresponded to 79.1% (34/43) of the total, with seven in state A, 16 in state B, and 11 in state C. The average I.Var. identified in the municipalities was 0.78 and considered high. The significant number of cities with I.Var. >0.7 (23/34; 70.6%) determined the high mean value identified (Fisher's test, *p* < 0.05). The number of cities/states with virulent strains did not fluctuate significantly, regardless of the percentage range of virulent strains. However, a significant minority of cities (4/34; 11.8%) had 40% or fewer of their virulent strains. Controversially, cities with 0–40% of the multi-virulent strains were predominant (24/34; 70.6%) in our study. This high number is because 100% of the municipalities in state C fall within this range (Table 7).

**Table 7:** Distribution of cities according to the percentage value of virulent and multi-virulent strains of *C. jejuni* isolated in municipalities located in three Brazilian states (A, B, and C).

**Percentage range of strains by city with the characteristics**	**No. of cities/ state**	**Cities with virulent strains [n (%)]**	**Cities with multi-virulent strains [n (%)]**
	**A (7)**	5 (71.4)^a^	1 (14.3)^a^
**100–70**	**B (16)**	9 (56.2)^a^	1 (6.3)^a^
	**C (11)**	3 (27.3)^a^	0^a^
	**Total (34)**	17 (50.0)^A^	2 (5.9)^A^
	**A (7)**	1 (14.3)^a^	4 (57.1)^a^
**70–40**	**B (16)**	5 (31.3)^a^	4 (25.0)^ab^
	**C (11)**	7 (63.6)^a^	0^b^
	**Total (34)**	13 (38.2)^A^	8 (23.5)^A^
	**A (7)**	1 (14.3)^a^	2 (28.6)^a^
**40–0**	**B (16)**	2 (12.5)^a^	11 (68.8)^ab^
	**C (11)**	1 (9.1)^a^	11 (100)^b^
	**Total (34)**	4 (11.8)^B^	24 (70.6)^B^

^ab^different letters in the same column indicate a difference within the same range. ^AB^different letters in the same column indicate a difference between the intervals (p < 0.05).

The I.Var. and the virulence and multi-virulence characteristics were evaluated for the 359 strains, considering the months from October to December 2017 and January and March to July 2018.

**Figure 5:**
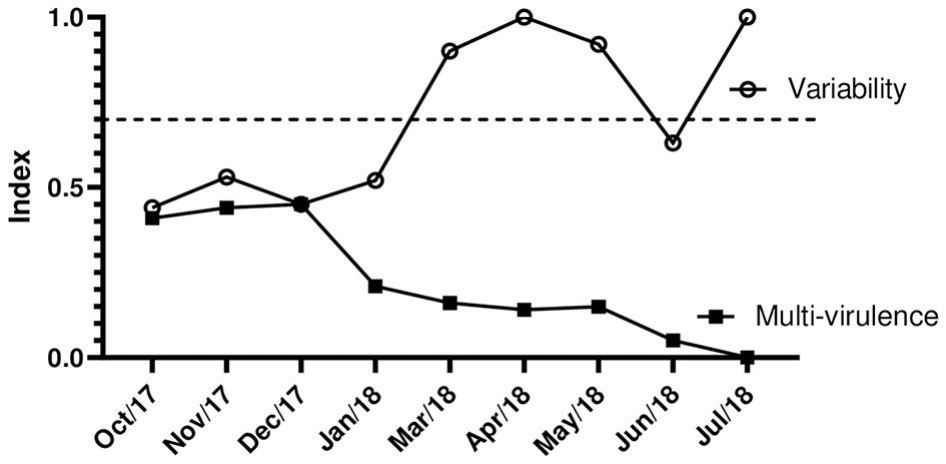
Indexes of variability and multi-virulence of *C. jejuni* over time of isolation. The dashed line (—) separates low and medium indexes from values of high genetic variability.

High I.Var. are directly related to low rates of multi-virulence due to the odds ratio (Table 7). Our study showed that we had the lowest rates of multi-virulence in the months from January to July 2018, with a decreasing characteristic over time. This determined the high genetic variability between the strains due to the diversity of virulence profiles. In the months from October to December 2017, there was greater balance and equivalence in these indexes (Figure 5).

In general, the months considered hot and humid (October to December) in the southern hemisphere determine the highest number of isolates associated with high rates of virulence and multi-virulence, indicating the seasonal characteristic of *C. jejuni* (Table 8).

**Table 8:** Frequency of virulence and multi-virulence of strains and profiles by seasonality.

**Month/year**	**Virulence [*****n*** **(%)]**		**Multi-virulence [*****n*** **(%)]**		**Total**		**OR I.Var.–I.M.^−1^**
	**Strains**	**Profiles**	**Strains**	**Profiles**	**Strains**	**Profiles**	
October/2017	73 (67.6)^ac, A^	26 (54.2)^ab, C^	44 (40.7)^a, B^	11 (22.9)^a, D^	108	48	1.16
November/2017	37 (82.2)^ad, A^	16 (66.7)^a, C^	20 (44.4)^a, B^	6 (25.0)^a, D^	45	24	1.43
December/2017	37 (78.7)^ad, A^	13 (61.9)^ab, C^	21 (44.7)^a, B^	2 (9.5)^a, D^	47	21	1.0
January/2018	41 (53.2)^bc, A^	15 (37.5)^b, C^	16 (20.8)^bc, B^	3 (7.5)^a, D^	77	40	4.12
March/2018	8 (42.1)^b, A^	6 (35.3)^ab, C^	3 (15.8)^bd, A^	3 (17.6)^a, C^	19	17	45.33
April/2018	4 (57.1)^bcd, A^	4 (57.1)^ab, C^	1 (14.3)^ad, A^	1 (14.3)^a, C^	7	7	∞
May/2018	8 (61.5)^bcd, A^	7 (58.3)^ab, C^	2 (15.4)^ad, B^	2 (16.7)^a, C^	13	12	66.0
June/2018	20 (52.6)^bc, A^	15 (62.5)^ab, C^	2 (5.3)^d, B^	2 (8.3)^a, D^	38	24	30.86
July/2018	1 (20.0)^b, A^	1 (20.0)^ab, C^	0^abd, A^	0^a, C^	5	5	∞

Different lowercase letters in the same column indicate a significant difference. AB: difference in virulence and multi-virulence of strains (line). CD: difference in virulence and multi-virulence of the profiles (line) (p < 0.05). OR, odds ratio; I.Var., variability index; I.M., multi-virulence index.

## 4. Discussion

Brazil, the world's largest exporter of chicken meat, encourages further research on *Campylobacter*, the main food-borne pathogen found in chicken meat, to acquire in-depth knowledge of the epidemiological and molecular characteristics of the strains present in the country.

In particular, there is probable selection pressure for *Campylobacter* due to adaptive characteristics to stress conditions that directly or indirectly influence their suitability, specifically in species adapted to the host but widely exposed to hostile environmental conditions, as is the case for *C. jejuni* (Costa and Iraola, 2019). The significant prevalence of strains that concomitantly present genes associated with adaptation to stress (309/359; 86.07%) (Table 2) suggests the existence of strains capable of survival in several niches. In parallel, this adaptive potential can facilitate its dissemination since there is an intensification of the survival rate under thermal, osmotic, and oxidative stress. Especially for *dnaJ*, which encodes heat shock proteins, this is particularly important for the survival of *Campylobacter* in foods that are kept under refrigeration or frozen during marketing, as well as during their preparation if they are undercooked (Baserisalehi and Bahador, 2011).

The high prevalence of the presence of the *hcp* gene (97.77%) linked to the category of secretion systems indicates the potential to cause more serious infections associated with bloody diarrhea (Kovács et al., 2020), since it is involved in the type VI secretion system (T6SS) that is responsible for carrying toxins into the environment and prokaryotic (Schwarz et al., 2010) or eukaryotic (Jani and Cotter, 2010) cells. The other genes related to the secretion system (*cdtABC*) had lower and similar frequencies (42–48%) in our study.

In general, the complete presence of the CDT genetic complex indicates the potential for the release of a functional cytotoxin that contributes to virulence, invasion, and cell adhesion and is essential for the release of interleukin-8 (IL-8), which contributes to the inflammatory response of the host mucosa as well as favoring the loss of integrity of the intestinal epithelium and cellular junctions and inducing apoptosis (Hickey et al., 2000).

The significant difference in the prevalence of genes linked to the CDT complex and the *hcp* gene may be related to the fact that, although they are important secretion systems in the pathogenesis of *C. jejuni*, both are encoded by independent regions of the genome and are therefore closely related (Asakura et al., 2007; Harrison et al., 2014). Similar to the CDT complex, genes linked to the invasion/colonization category had a concomitant prevalence lower than expected (63.79%) when compared to other studies conducted in Brazil (Melo et al., 2013, 2019). Immune evasion in some strains is an important factor reported in other studies, especially in the initial stages of invasion/colonization, facilitating long-term maintenance in the host through the establishment of chronic infection (Mikonranta et al., 2015; Petrovska et al., 2017).

It is estimated that 25–50% of cases of GBS can be associated with previous campylobacteriosis (Willison et al., 2016). The presence of the *neuA* and *cstII* genes demonstrated a significant relationship with the production of lipopolysaccharides (LOS) from the *Campylobacter* cell wall involved in the development of the syndrome (Melo et al., 2017; Neal-McKinney et al., 2018), and these genes were identified in 88.86% and 77.44% of the strains, respectively, in our study. These values are alarming due to their superiority compared to the findings in the literature (Amon et al., 2012; Melo et al., 2017). However, despite a large number of isolates with the potential risk of developing post-infection neuropathy, the host's immune system (humoral and cellular immunity) is primarily responsible for the development of GBS.

The joint prevalence of genes related to the biofilm formation/motility virulence category was 182/359 (50.7%). The association of both characteristics in our study was due to the physiological involvement identified for the *luxS* and *flaA* genes in *C. jejuni* associated with the complexity of the biofilm formation process that can be reduced by up to 57% in strains that do not share both genetic factors (Plummer, 2012). The high capacity to form biofilms in production environments is quite evident for *C. jejuni* and involves several intrinsic and extrinsic mechanisms (Melo et al., 2017). However, especially for strains that do not share the biofilm/motility category (177/359; 49.3%), the production of biomass can be significantly compromised (Plummer, 2012), which can facilitate the control of these pathogens by the hygiene processes used by industry.

Considering the clusters identified in the 124 profiles, the significance of cluster A16, which includes virulent (57) and multi-virulent (14/57) profiles, is evident (Table 3). This sets up the discussion aimed at the virulence and multi-virulence indices identified from different perspectives.

We observed a similarity in the number of establishments and municipalities that presented more than 70% of their strains classified as virulent (21/46 and 17/43, respectively), of which these strains were multi-virulent in two cities/establishments; these are significant values compared to the lowest interval (Tables 5, 7). In a broader analysis, it became clear that these locations do not include state C in a significant way (Figure 2) but mainly state A (Figure 3), whose strains had a more dispersed origin in the territory but with a convergence of the indices of virulence and multi-virulence to the central and metropolitan regions of the state. It is likely that this state concentrates the largest arsenal of *C. jejuni* with a higher evolutionary level in comparison to other states involved in the study and is, therefore, the main hotspot for the maintenance of virulent strains since this species tends to acquire a greater number of virulence genes over time (Datta et al., 2003).

At the temporal level, it is evident that the quantitative distribution (Table 8), the virulent, and, mainly, the multi-virulent character of the strains (Figure 5) present a seasonal pattern so that we identify the higher rates in the hottest and most humid months of the year (October to December). This behavior was also recorded in a study carried out on *C. jejuni* isolated from chicken carcasses that showed a higher prevalence and virulence in the summer compared to the winter (Kim et al., 2019). The analysis of the variability indices showed discrepancies in the values found in the general analysis of the data and at the state level (<0.7) with the values obtained by sample, by establishment, and by municipality (>0.7). The high variability between strains isolated from the same sample (I.Var. = 0.82) indicates the existence of different genotypes of *C. jejuni* coexisting within the same host organism. In fact, this type of adaptation to sub-niches or common niches in the same host is proposed in *C. jejuni*, where up to 10 different genotypes have already been reported in a single chicken carcass (Colles et al., 2008). This shows that different strains, especially emerging ones (11), with different degrees of complexity and virulence can cohabit with the same host in a commensal manner. In parallel, the similarity in I.Var. found in the strains considering establishments and municipalities (average >0.7) was expected. This is because we had two different establishments present in only three of the 43 investigated cities; for the remainder, the number of strains per municipality and establishment was equivalent. The high variability may be related to the environmental pressure that tends to select different profiles due to the different management and conduct adopted in each establishment and, in general, considering the intrinsic differences of the productive process itself, which involve stages in different degrees of stress that are tolerable or not to the different strains (Kudirkiene et al., 2011).

At the same time, *C. jejuni* has high genetic plasticity due to its high potential to carry out horizontal gene transfer and recombination since the bacterium is naturally competent for the uptake and transformation of DNA, which favors the diversity of virulence profiles (Burnham and Hendrixson, 2019). Paradoxically, the analysis of I.Var. in the total sampling and its distribution at the state level shows lower values due to the concentration of strains in the same group (total or by state). These lower values occurred due to the existence of a large number of strains grouped in a few profiles classified as virulent and multi-virulent, which represents an evolutionary trend in *C. jejuni* (Melo et al., 2019). The distribution of I.Var. over time showed an inverse association with the multi-virulence index (odds ratio >1.0) (Table 8). This fact is configured by elevated I.Var. in months with low rates of MV strains. In addition, low variability occurred in the months that include the summer season, which had the highest rates of MV strains. The steep drop in I.Var. associated with the multi-virulence of strains in hot and humid periods of the year is consistent with the significant increases recorded worldwide in enteritis caused by *Campylobacter*, associated with a surprising increase in the severity of cases (Casey et al., 2017).

## 5. Conclusion

Considering the complexity of the epidemiology of *C. jejuni*, our results may contribute to strategies for its control. The gene profile demonstrates that the studied strains have the potential for high adaptation to hostile environmental situations; however, on the other hand, they have a lower frequency of genes linked to biofilm formation, indicating that adequate hygiene processes can be strategic for their control. Finally, the high levels of virulence, especially in summer and in state A, suggest the need to adopt control measures converging with these findings.

## Data availability statement

The original contributions presented in the study are included in the article/supplementary material, further inquiries can be directed to the corresponding author.

## Author contributions

Conceived and designed the experiments: PABMP and DAR. Performed the experiments: PABMP, ECAL, ASMB, MdVF, ABGB, and EPM. Analyzed the data: PABMP, RTdM, ALG, and GPM. Contributed reagents/materials/analysis tools: DAR, PMA, FB, and TFP. Wrote the paper: PABMP and RTdM.

## References

[B1] ABPA (2023). Relatório Anual 2023, 2023. Available online at: https://abpa-br.org/wp-content/uploads/2023/04/Relatorio-Anual-2023.pdf (accessed February 22, 2023).

[B2] Amon P. Klein D. Springer B. Jelovcan S. Sofka D. Hilbert F.. (2012). Analysis of *Campylobacter jejuni* isolates of various sources for loci associated with Guillain-Barré syndrome. Eur. J. Microbiol. Immunol. 2, 20–23. 10.1556/EuJMI.2.2012.1.4 24611117 PMC3933986

[B3] Asakura M. Samosornsuk W. Taguchi M. Kobayashi K. Misawa N. Kusumoto M.. (2007). Comparative analysis of cytolethal distending toxin (cdt) genes among *Campylobacter jejuni, C. coli* and *C. fetus* strains. Microb Pathog. 42, 174–183. 10.1016/j.micpath.2007.01.005 17353111

[B4] Baserisalehi M. Bahador N. (2011). Chemotactic behavior of *Campylobacter* spp. in function of different temperatures (37 C and 42 C). Anaerobe. 17, 459–462. 10.1016/j.anaerobe.2011.06.010 21757020

[B5] Biswas D. Hannon S. J. Townsend H. G. Potter A. Allan B. J. (2011). Genes coding for virulence determinants of *Campylobacter jejuni* in human clinical and cattle isolates from Alberta, Canada, and their potential role in colonization of poultry. Int Microbiol. 14, 25–32. 10.2436/20.1501.01.132 22015699

[B6] Bolton D. J. (2015). *Campylobacter* virulence and survival factors. Food microbiology. 48, 99–108. 10.1016/j.fm.2014.11.017 25790997

[B7] Burnham P. M. Hendrixson D. R. (2019). *Campylobacter jejuni*: collective components promoting a successful enteric lifestyle. Nat. Rev. Microbiol. 16, 551–565. 10.1038/s41579-018-0037-9 29892020

[B8] Casey E. Fitzgerald E. Lucey B. (2017). Towards understanding clinical *Campylobacter* infection and its transmission: time for a different approach?. Br. J. Biomed. Sci. 74, 53–64. 10.1080/09674845.2017.1291205 28367739

[B9] CDC (2019). Preliminary Incidence and Trends of Infections with Pathogens Transmitted Commonly Through Food — Foodborne Diseases Active Surveillance Network, 10 U.S. Sites, 2016–2019 MMWR Morb Mortal. Available online at: https://www.cdc.gov/foodnet/reports/prelim-data-intro-2019.html (accessed March 30, 2019).

[B10] Colles F. M. Dingle K. E. Cody A. J. Maiden M. C. J. (2008). Comparison of *Campylobacter* populations in wild geese with those in starlings and free-range poultry on the same farm. Appl. Environ. Microbiol. 74, 3583–3590. 10.1128/AEM.02491-07 18390684 PMC2423018

[B11] Costa D. Iraola G. (2019). Pathogenomics of emerging *Campylobacter* species. Clin. Microbiol. Rev. 32, e00072–e00018. 10.1128/CMR.00072-18 31270126 PMC6750134

[B12] Datta S. Niwa H. Itoh K. (2003). Prevalence of 11 pathogenic genes of *Campylobacter jejuni* by PCR in strains isolated from humans, poultry meat and broiler and bovine faeces. J. Med. Microbiol. 52, 345–348. 10.1099/jmm.0.05056-0 12676874

[B13] EFSA (2020). Update and Review of Control Options for Campylobacter in Broilers At Primary Production, EFSA Journal. Available online at: https://www.efsa.europa.eu/en/efsajournal/pub/6090 (accessed March 15, 2020). 10.2903/j.efsa.2020.6090 PMC7448041 32874298

[B14] Elvers K. T. Park S. F. (2012). Quorum sensing in *Campylobacter jejuni*: detection of a luxS encoded signalling molecule. Microbiology. 148, 1475–1481. 10.1099/00221287-148-5-1475 11988522

[B15] Hänel I. Müller J. Müller W. Schulze F. (2004). Correlation between invasion of Caco-2 eukaryotic cells and colonization ability in the chick gut in *Campylobacter jejuni*. Vet. Microbiol. 2, 75–82. 10.1016/j.vetmic.2004.04.004 15172689

[B16] Harrison J. W. Dung T. T. N. Siddiqui F. Korbrisate S. Bukhari H. Tra M. P. V.. (2014). Identification of possible virulence marker from *Campylobacter jejuni* isolates. Emerg. Infect. Dis. 20, 1026–1029. 10.3201/eid2006.130635 24856088 PMC4036754

[B17] Hickey T. E. McVeigh A. L. Scott D. A. Michielutti R. E. Bixby A. Carroll S. A.. (2000). *Campylobacter jejuni* cytolethal distending toxin mediates release of interleukin-8 from intestinal epithelial cells. Infect. Immun. 68, 6535–6541. 10.1128/IAI.68.12.6535-6541.2000 11083762 PMC97747

[B18] ISO (2017). - Microbiology of Food and Animal Feeding Stuffs – Horizontal Method for Detection and Enumeration of Campylobacter spp. – Part 1: Detection Method (ISO 10272-1, 2006). Available online at: https://www.iso.org/standard/63228.html (accessed May 5, 2020).

[B19] Jani A. J. Cotter P. A. (2010). Type VI secretion: not just for pathogenesis anymore. Cell Host. Microbe. 8, 2–6. 10.1016/j.chom.2010.06.012 20638635 PMC2913581

[B20] Kaakoush N. O. Castaño-Rodríguez N. Mitchell H. M. Man S. M. (2015). Global epidemiology of *Campylobacter* infection. Clin. Microbiol. Rev. 28, 687–720. 10.1128/CMR.00006-15 26062576 PMC4462680

[B21] Kim J. Park H. Kim J. Kim J. H. Jung J. I. Cho S.. (2019). Comparative analysis of aerotolerance, antibiotic resistance, and virulence gene prevalence in *Campylobacter jejuni* isolates from retail raw chicken and duck meat in South Korea. Microorganisms. 7, 1–13. 10.3390/microorganisms7100433 31658662 PMC6843641

[B22] Kovács J. K. Cox A. Schweitzer B. Maróti G. Kovács T. Fenyvesi H.. (2020). Virulence traits of inpatient *Campylobacter jejuni* isolates, and a transcriptomic approach to identify potential genes maintaining intracellular survival. Microorganisms. 8, 531. 10.3390/microorganisms8040531 32272707 PMC7232156

[B23] Krumperman P. H. (1983). Multiple antibiotic resistance indexing of *Escherichia coli* to identify high-risk sources of fecal contamination of foods. Appl. Environ. Microbiol. 46, 165–170. 10.1128/aem.46.1.165-170.1983 6351743 PMC239283

[B24] Kudirkiene E. Bunevičien,e J. Brøndsted L. Ingmer H. Olsen J. E. Malakauskas M.. (2011). Evidence of broiler meat contamination with post-disinfection strains of *Campylobacter jejuni* from slaughterhouse. Int. J. Food Microbiol. 145, S116–S120. 10.1016/j.ijfoodmicro.2010.06.024 20647156

[B25] Llarena A. K. Taboada E. Rossi M. (2017). Whole-genome sequencing in epidemiology of *Campylobacter jejuni* infections. J. Clin. Microbiol. 55, 1269–1275. 10.1128/JCM.00017-17 28249998 PMC5405246

[B26] MAPA (2016). Instrução Normativa n° 20, de 21 de outubro de 2016. Brasil, 2016^a^. Available online at: http://www.agricultura.gov.br/assuntos/inspecao/produtos-animal/controle-depatogenos/arquivos-controle-de-patogenos/SalmonellaIN202016Salmonella.pdf (accessed May 10, 2016).

[B27] Martínez I. Mateo E. Churruca E. Girbau C. Alonso R. Fernandez-Astorga A.. (2006). Detection of *cdtA, cdtB*, and *cdtC* genes in *Campylobacter jejuni* by multiplex PCR. Int. J. Med. Microbiol. 296, 45–48. 10.1016/j.ijmm.2005.08.003 16423686

[B28] Melo R. T. Grazziotin A. L. Júnior E. C. V. Prado R. R. Mendonça E. P. Monteiro G. P.. (2019). Evolution of *Campylobacter jejuni* of poultry origin in Brazil. Food Microbiol. 82, 489–496. 10.1016/j.fm.2019.03.009 31027810

[B29] Melo R. T. Mendonça E. P. Monteiro G. P. Siqueira M. C. Pereira C. B. Peres P. A. B. M.. (2017). Intrinsic and extrinsic aspects on *Campylobacter jejuni* biofilms. Front. Microbiol. 8, 1332. 10.3389/fmicb.2017.01332 28769900 PMC5513903

[B30] Melo R. T. Nalevaiko P. C. Mendonça E. P. Borges L. W. Fonseca B. B. Beletti M. E.. (2013). *Campylobacter jejuni* strains isolated from chicken meat harbour several virulence factors and represent a potential risk to humans. Food Control. 33, 227–231. 10.1016/j.foodcont.2013.02.032

[B31] Mikonranta L. Mappes J. Laakso J. Ketola T. (2015). Within-host evolution decreases virulence in an opportunistic bacterial pathogen. BMC Evol. Biol.15, 165. 10.1186/s12862-015-0447-5 26282271 PMC4539714

[B32] Neal-McKinney J. M. Liu K. C. Jinneman K. C. Wu W. H. Rice D. H. (2018). Whole genome sequencing and multiplex qPCR methods to identify *Campylobacter jejuni* encoding *cst-II* or *cst-III* sialyltransferase. Front. Microbiol. 9, 1–8. 10.3389/fmicb.2018.00408 29615986 PMC5865068

[B33] Omurtag I. Paulsen P. Hilbert F. Smulders F. J. M. (2013). The risk of transfer of foodborne bacterial hazards in Turkey through the consumption of meat; risk ranking of muscle foods with the potential to transfer *Campylobacter* spp. Food Secur. 5, 117–127. 10.1007/s12571-012-0230-z

[B34] Petrovska L. Tang Y. Jansen van Rensburg M. J. Cawthraw S. Nunez J. Sheppard S. K. (2017). Genome reduction for niche association in *Campylobacter hepaticus*, a cause of spotty liver disease in poultry. Front. Cell Infect. Microbiol. 11, 354. 10.3389/fcimb.2017.00354 28848714 PMC5554493

[B35] Plummer P. J. (2012). LuxS and quorum-sensing in *Campylobacter*. Front. Cellular Infect. Microbiol. 2, 1–22. 10.3389/fcimb.2012.00022 22919614 PMC3417632

[B36] Rodrigues C. S. Armendaris P. M. Haddad C. V. G. C. Melo J. P. A. C. B. (2021). Prevalence of *Campylobacter* spp. in chicken carcasses in slaughterhouses from south of Brazil. Curr. Microbiol. 78, 2242–2250. 10.1007/s00284-021-02478-w 33830320

[B37] Schwarz S. Hood R. D. Mougous J. D. (2010). What is type VI secretion doing in all those bugs?. Trends Microbiol. 18, 531–537. 10.1016/j.tim.2010.09.001 20961764 PMC2991376

[B38] Serichantalergs O. Wassanarungroj P. Khemnu N. Poly F. Guerry P. Bodhidatta L.. (2020). Distribution of genes related to Type 6 secretion system and lipooligosaccharide that induced ganglioside mimicry among *Campylobacter jejuni* isolated from human diarrhea in Thailand. Gut. Pathog. 12, 1–10. 10.1186/s13099-020-00357-6 32308743 PMC7146907

[B39] Singh A. Nisaa K. Bhattacharyya S. Mallick A. I. (2019). Immunogenicity and protective efficacy of mucosal delivery of recombinant hcp of *Campylobacter jejuni* Type VI secretion system (T6SS) in chickens. Mol. Immunol. 111, 182–197. 10.1016/j.molimm.2019.04.016 31078054

[B40] Ungureanu V. A. Stratakos A. C. Gundogdu O. Stef L. Pet I. Pet E.. (2019). Virulence of a T6SS *Campylobacter jejuni* chicken isolate from North Romania. BMC Res. Notes. 12, 1–7. 10.1186/s13104-019-4201-8 30922352 PMC6437841

[B41] Willison H. J. Jacobs B. C. Van Doorn P. A. (2016). Guillain-barre syndrome. The Lancet 388, 717–727. 10.1016/S0140-6736(16)00339-1 26948435

[B42] Zheng J. Meng J. H. Zhao S. H. Singh R. Song W. X. (2006). Adherenceto and invasion of human intestinal epithelial cells by *Campylobacter jejuni* and *Campylobacter coli* isolates from retail meat products. J. Food Prot. 69, 768–774. 10.4315/0362-028X-69.4.768 16629018

